# The Importance of immunonutrition in children

**DOI:** 10.1186/1824-7288-40-S1-A40

**Published:** 2014-08-11

**Authors:** Roberto Berni Canani, Lorella Paparo, Rosita Aitoro, Yelenia Maddalena, Simona Caprio, Antonio Amoroso, Carmen di Scala, Ludovica Leone, Rita Nocerino

**Affiliations:** 1 Department of Translational Medical Sciences and European Laboratory for the Investigation of Food Induced Diseases, University of Naples “Federico II”, Naples, Italy

## 

A healthy immune system is essential for prevention and recovery in many pediatric illnesses. During last decade, the role of nutrition beyond providing the calories and the macro- and micronutrients for body growth has been well established and clinically proven. Many nutrients have a tremendous potential to modulate directly or indirectly, through a regulation of gut microbiota composition, the development and function of innate and acquired immunity. The potential to modulate the activity of the immune system by interventions with specific nutrients is termed immunonutrition. When we prescribe a particular diet it is important to think that nutrients are not only factors able to influence body growth, but they are also a crucial driving force leading to body health through a regulation of immune system. Within the same category of nutrients it is possible to observe different effects on immune system. As example of this, comparing iso-energetic and iso-proteic doses of different mammalian milks (human milk, donkey milk and bovine milk) we have recently demonstrated, in an animal model, significantly different immunoregulatory and antioxidant properties. These effects are at least in part related to a modification of gut microbiota composition and function, and are able to modulate energy balance, glucose and lipid metabolism. We can modify the immunonutritional properties of a particular food. We have recently demonstrated the possibility through a dietary supplementation with fermented bovine milk with a selected probiotic strain (*Lactobacillus paracasei* CBA-L74) to significantly reduce the number of common winter infectious diseases in school-age children. This preventive effect derives from a complex network of different mechanisms of action: modulation of gut microbiota composition, stimulation of short chain fatty acids production, stimulation of innate immunity (alpha and beta-defensins, and cathelicidin LL-37 production), stimulation of acquired immunity (secretory IgA), modulation of gut permeability, modulation of epithelial cell growth and differentiation. Significant progress at identifying the gut microbiome has led to a better understanding of the interactions between them and our organs and tissues. Probiotics, while not considered a nutrient, are certainly became a even more frequent component of children diet. The roles that ingested organisms may play in atopic diseases are now potential targets of prevention and treatment.

